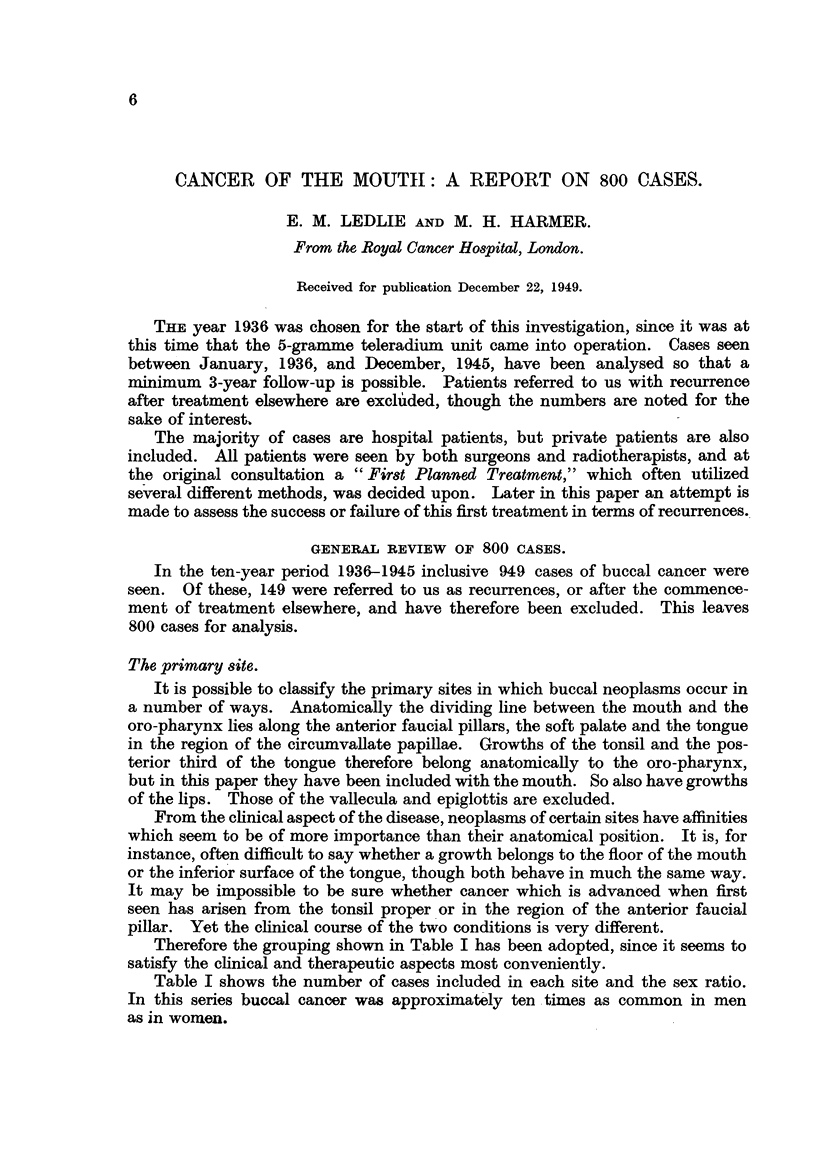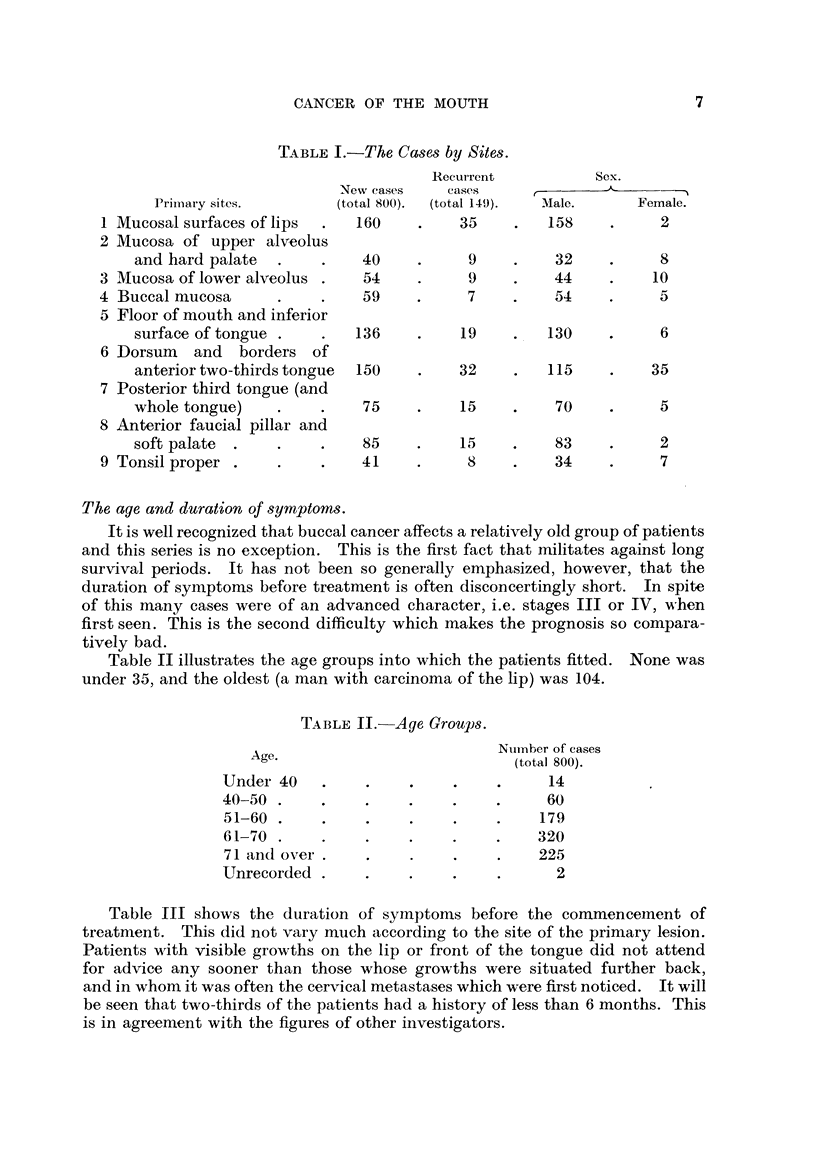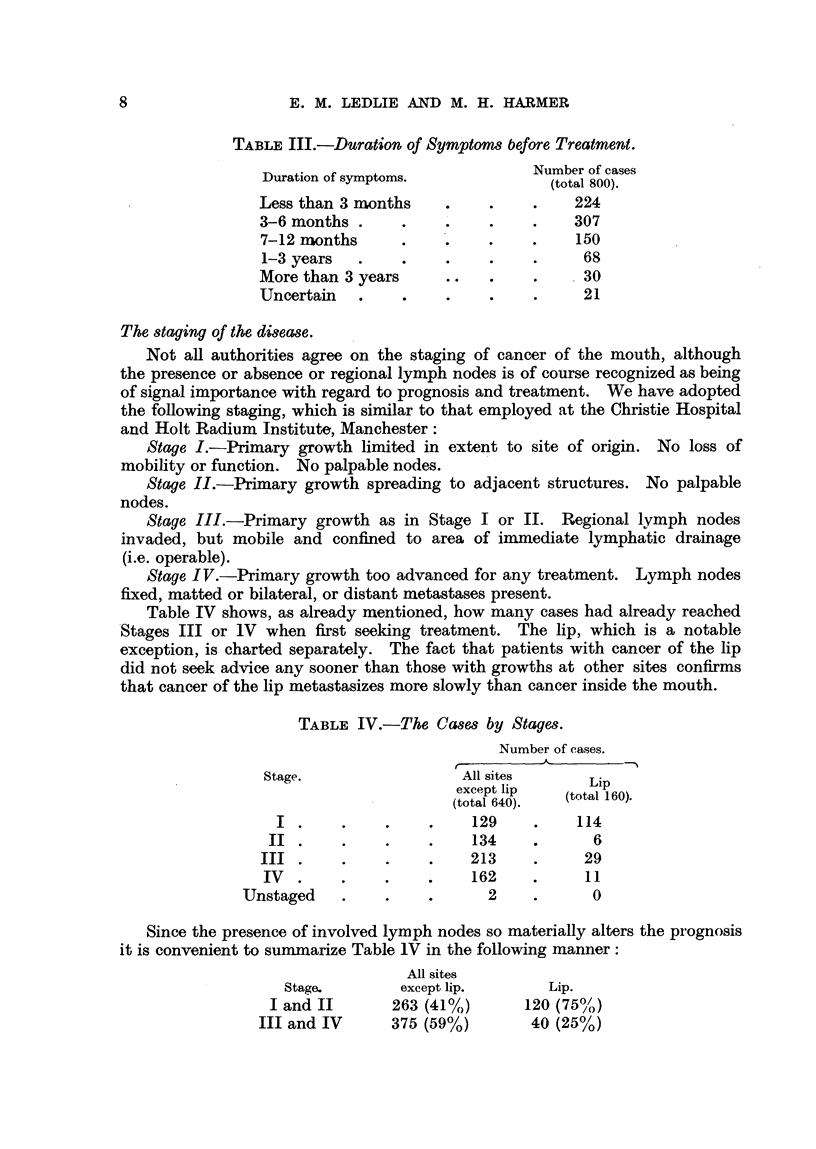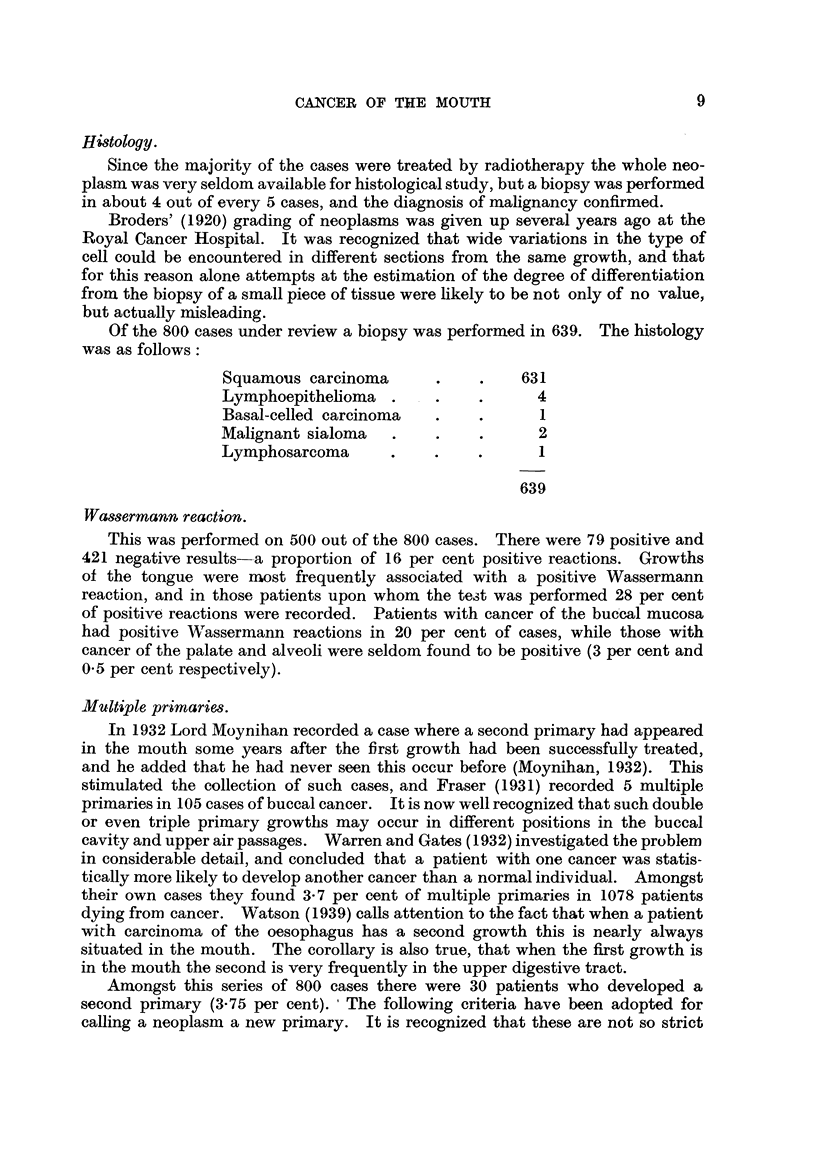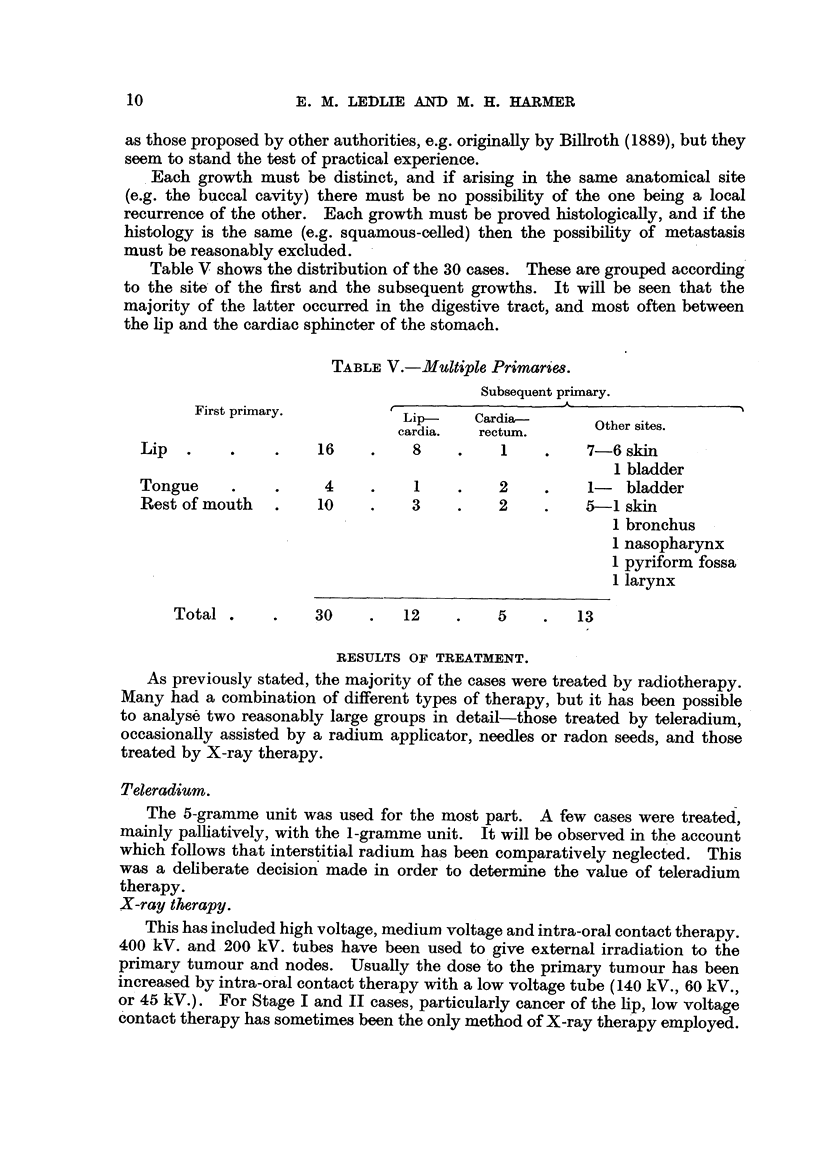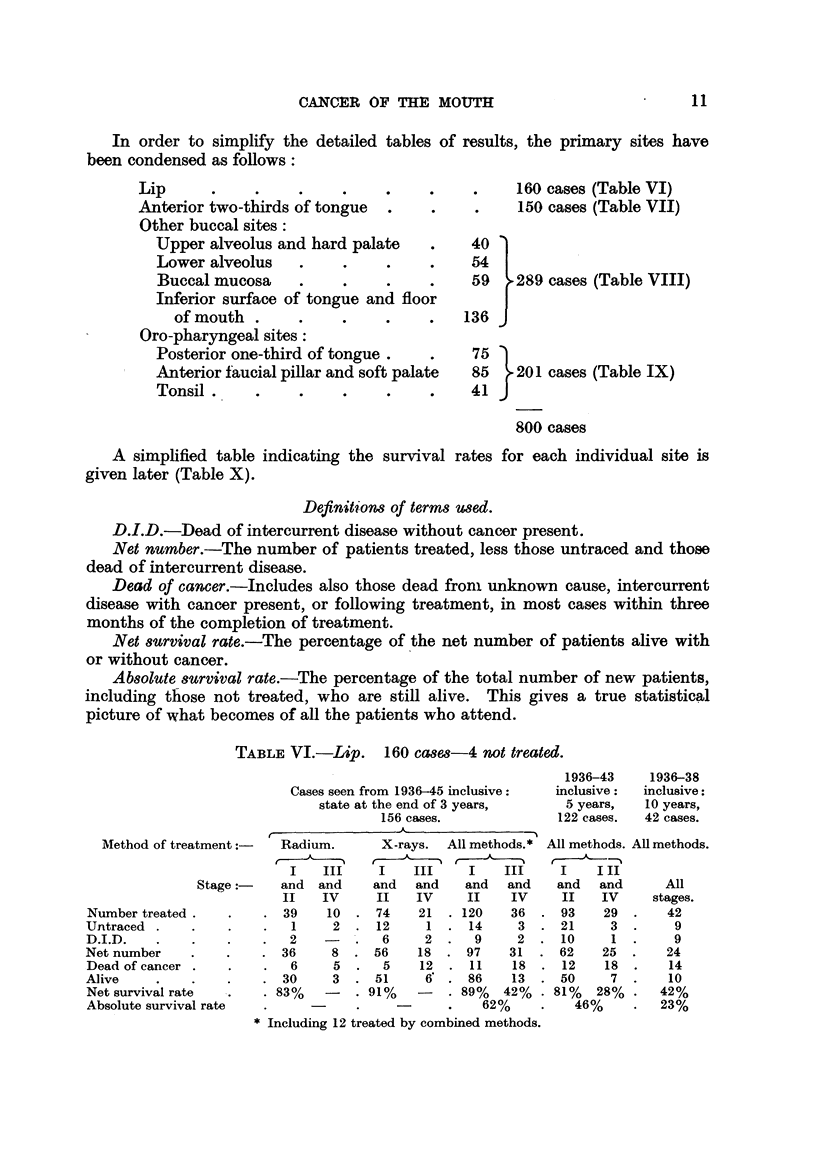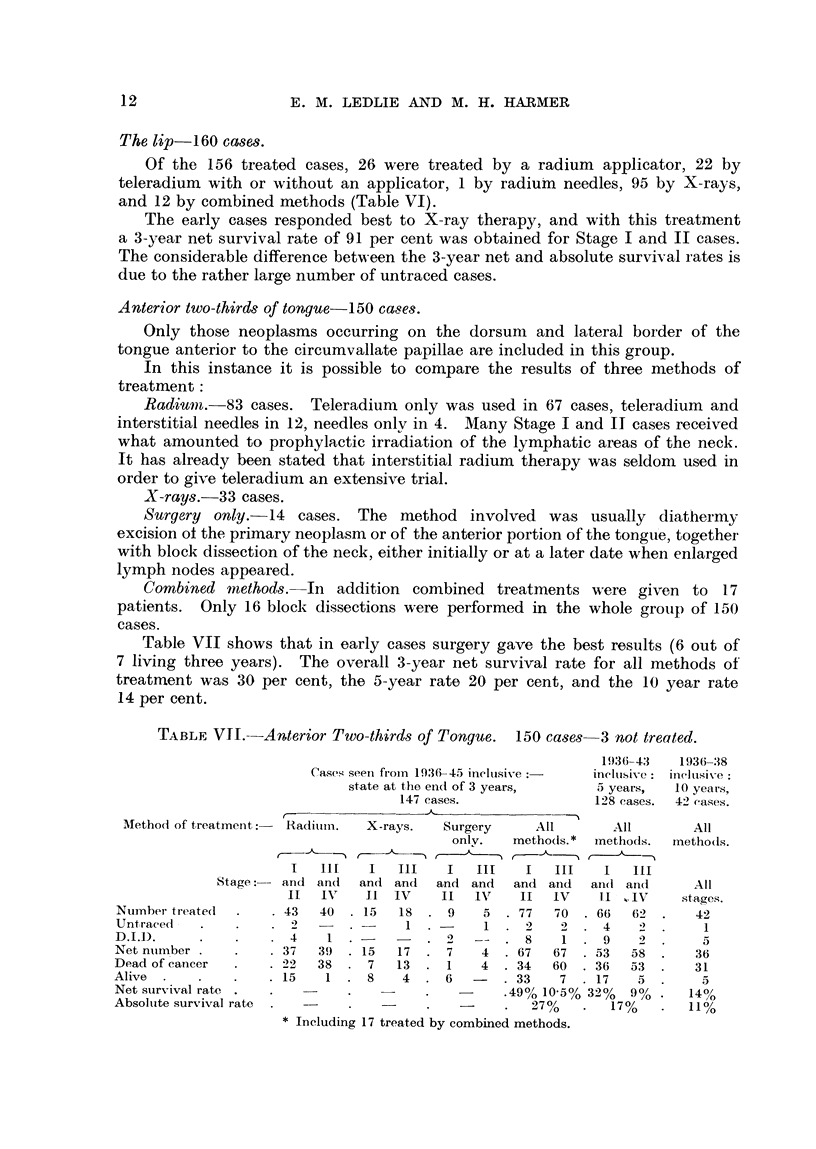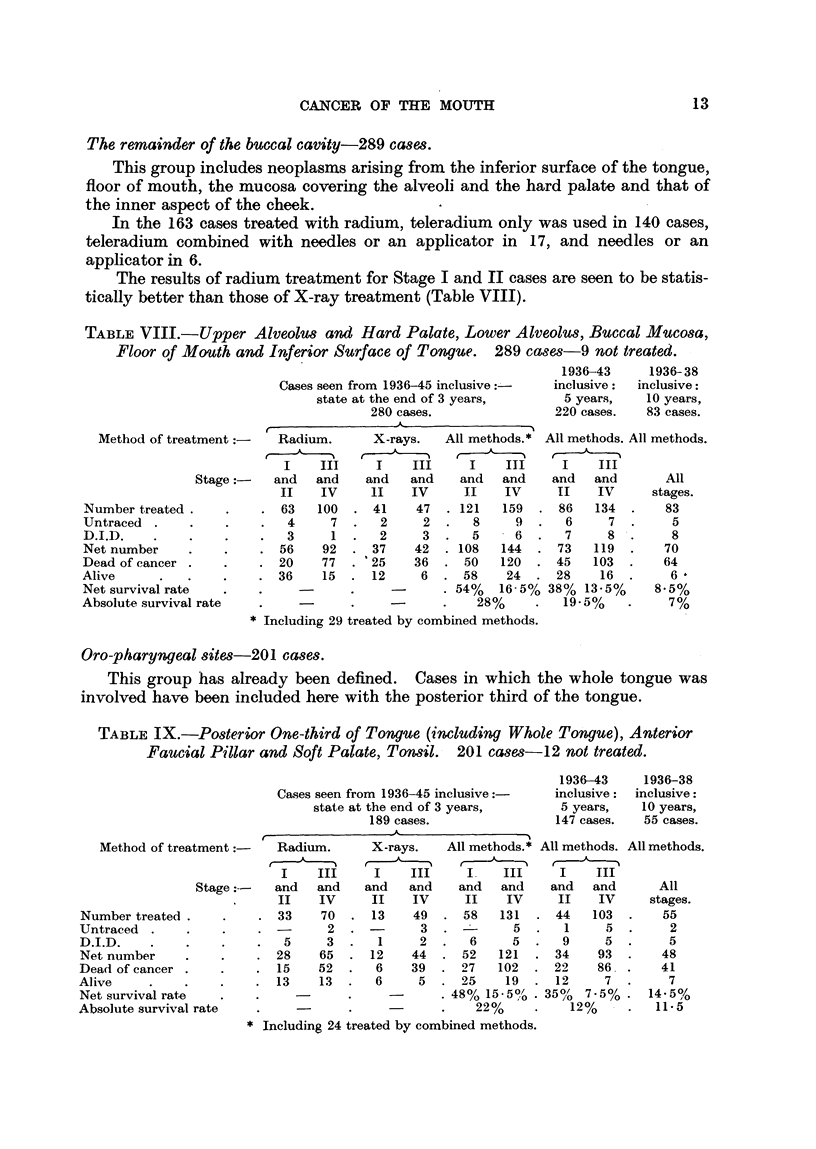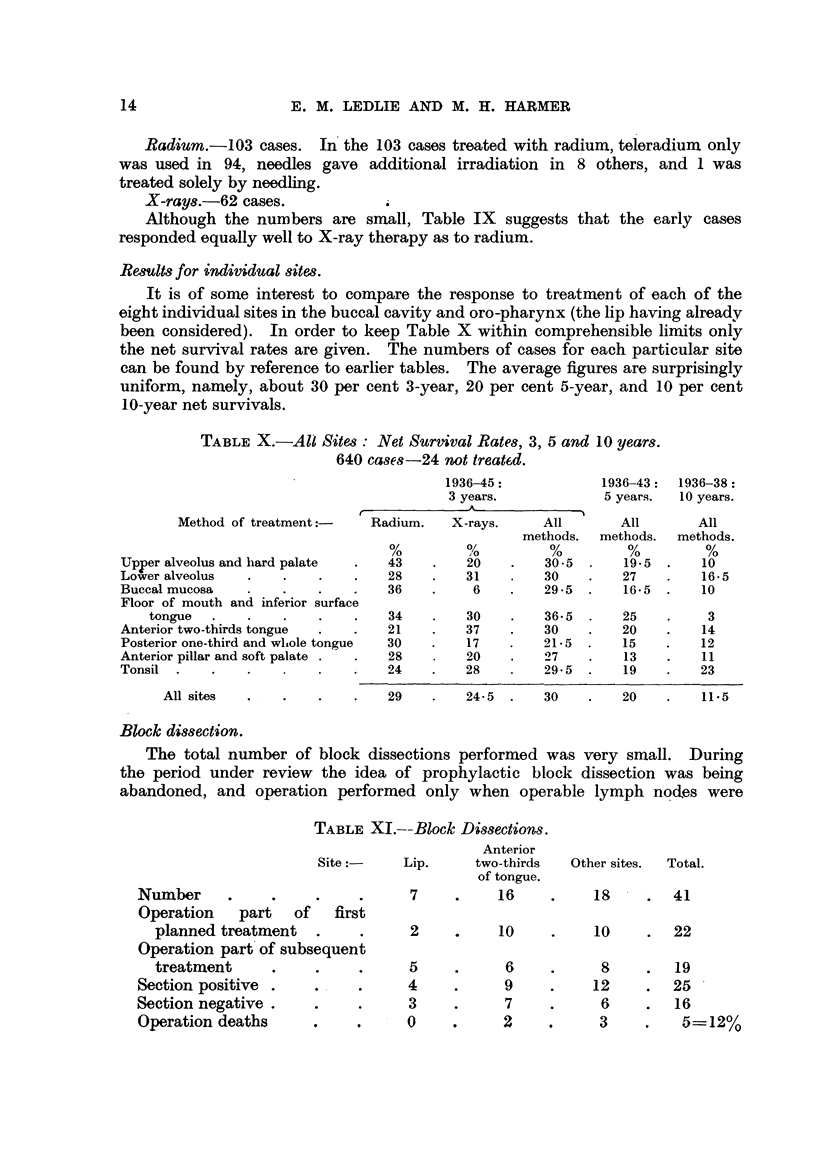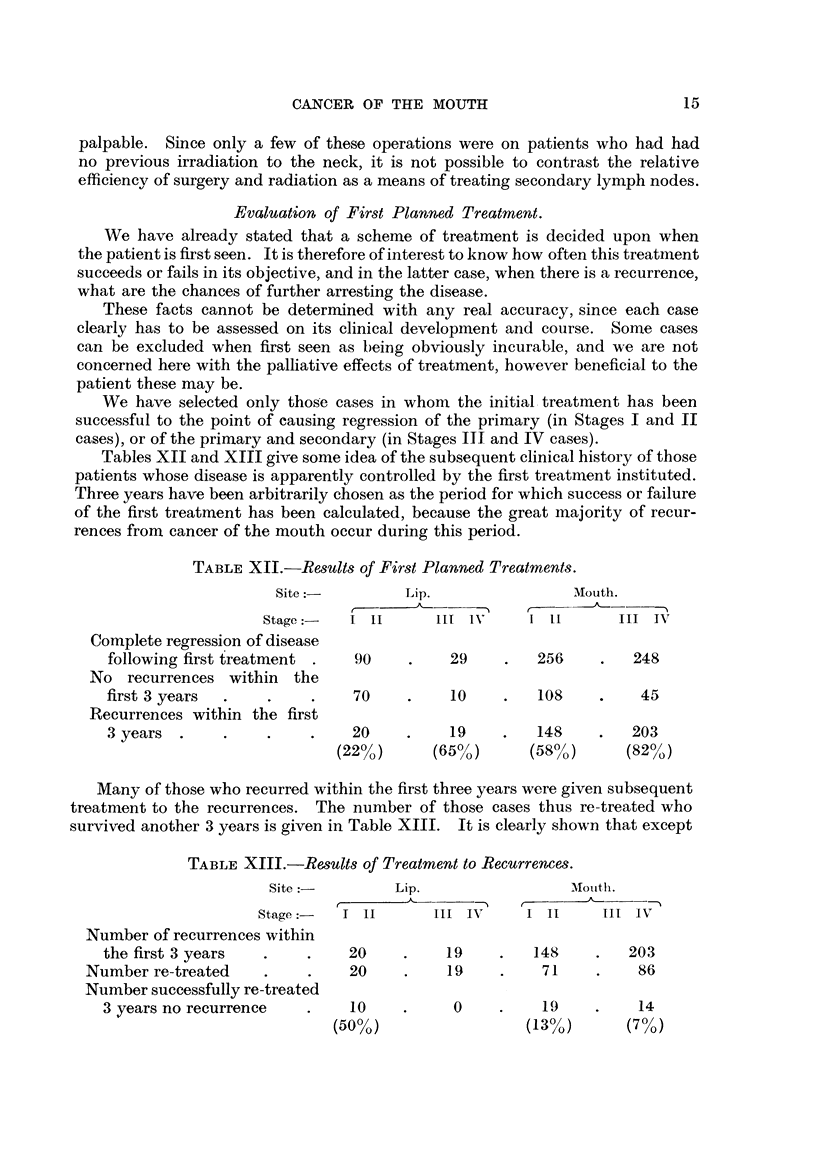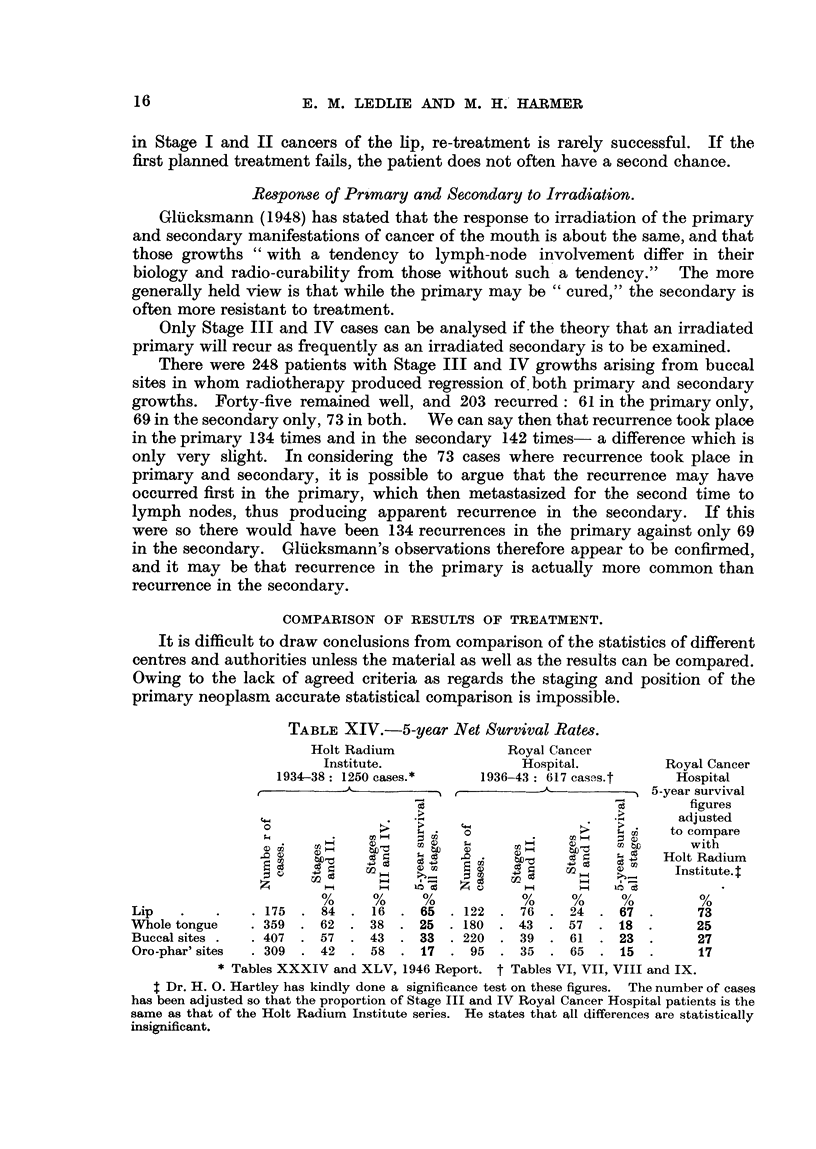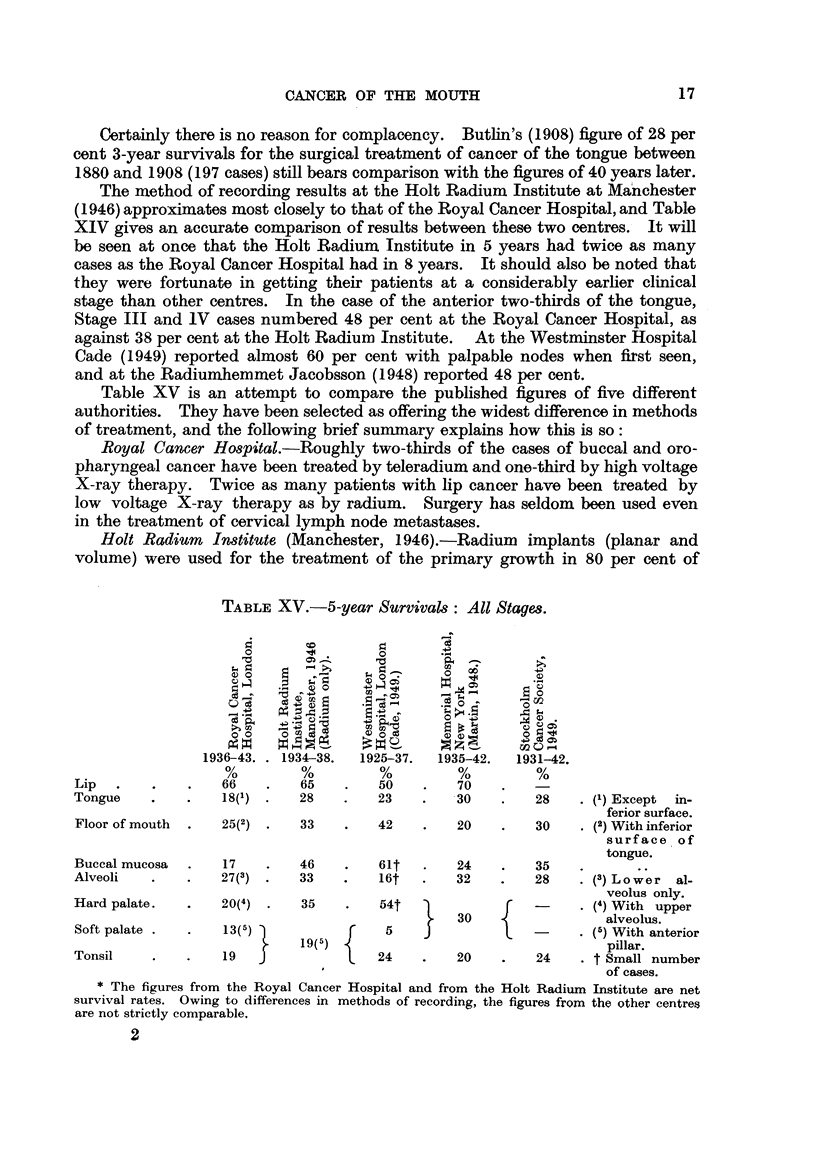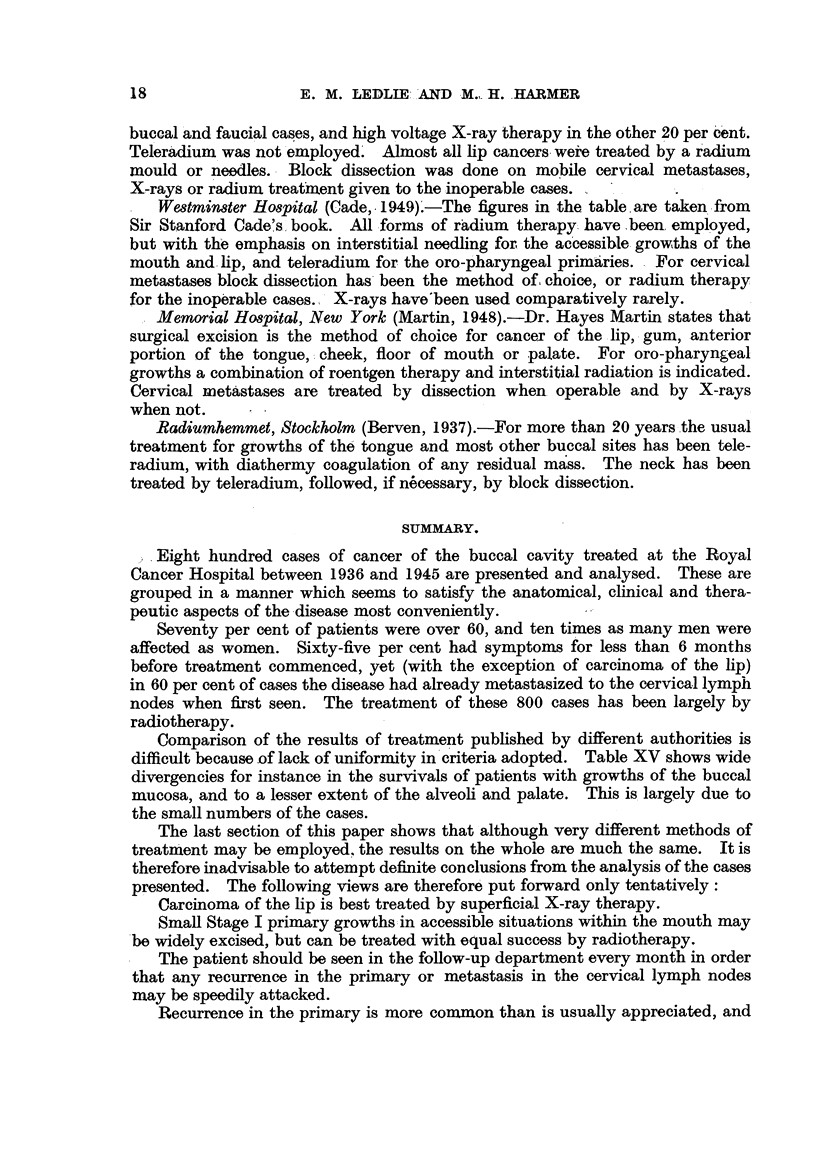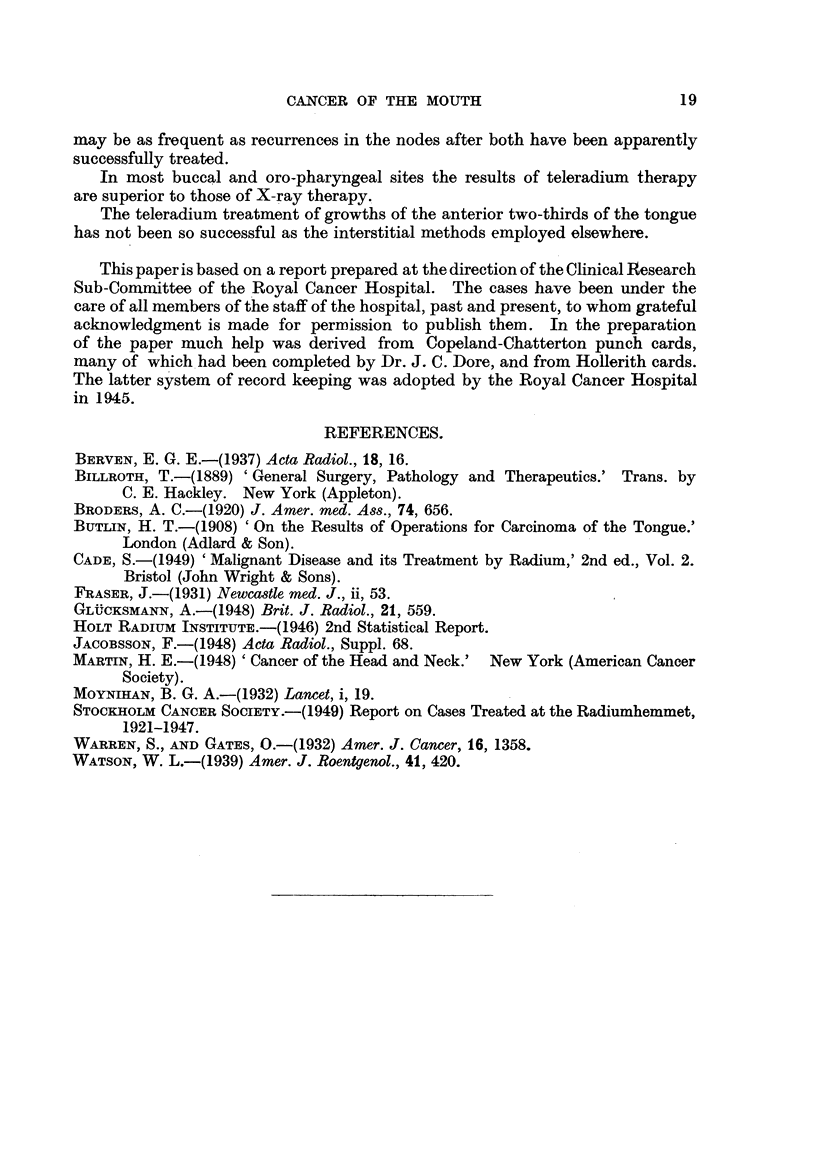# Cancer of the Mouth: A Report on 800 Cases

**DOI:** 10.1038/bjc.1950.2

**Published:** 1950-03

**Authors:** E. M. Ledlie, M. H. Harmer


### CANCER OF THE MOUTH: A REPORT ON 800 CASES.

E. M. LEDLIE AND M. H. HARMER.

*From the Royal Cancer Hospital, London.*

Received for publication December 22, 1949.

THE year 1936 was chosen for the start of this investigation, since it was at this time that the 5-gramme teleradium unit came into operation. Cases seen between January, 1936, and December, 1945, have been analysed so that a minimum 3-year follow-up is possible. Patients referred to us with recurrence after treatment elsewhere are excluded, though the numbers are noted for the sake of interest.

The majority of cases are hospital patients, but private patients are also included. All patients were seen by both surgeons and radiotherapists, and at the original consultation a "*First Planned Treatment*," which often utilized several different methods, was decided upon. Later in this paper an attempt is made to assess the success or failure of this first treatment in terms of recurrences.

### GENERAL REVIEW OF 800 CASES.

In the ten-year period 1936–1945 inclusive 949 cases of buccal cancer were seen. Of these, 149 were referred to us as recurrences, or after the commencement of treatment elsewhere, and have therefore been excluded. This leaves 800 cases for analysis.

### *The primary site.*

It is possible to classify the primary sites in which buccal neoplasms occur in a number of ways. Anatomically the dividing line between the mouth and the oro-pharynx lies along the anterior faucial pillars, the soft palate and the tongue in the region of the circumvallate papillae. Growths of the tonsil and the posterior third of the tongue therefore belong anatomically to the oro-pharynx, but in this paper they have been included with the mouth. So also have growths of the lips. Those of the vallecula and epiglottis are excluded.

From the clinical aspect of the disease, neoplasms of certain sites have affinities which seem to be of more importance than their anatomical position. It is, for instance, often difficult to say whether a growth belongs to the floor of the mouth or the inferior surface of the tongue, though both behave in much the same way. It may be impossible to be sure whether cancer which is advanced when first seen has arisen from the tonsil proper or in the region of the anterior faucial pillar. Yet the clinical course of the two conditions is very different.

Therefore the grouping shown in Table I has been adopted, since it seems to satisfy the clinical and therapeutic aspects most conveniently.

Table I shows the number of cases included in each site and the sex ratio. In this series buccal cancer was approximately ten times as common in men as in women.

TABLE I.—*The Cases by Sites.*

| Primary sites. | New cases (total 800). | Recurrent cases (total 149). | Sex. Male. | Female. |
|---|---|---|---|---|
| 1 Mucosal surfaces of lips | 160 | 35 | 158 | 2 |
| 2 Mucosa of upper alveolus and hard palate | 40 | 9 | 32 | 8 |
| 3 Mucosa of lower alveolus | 54 | 9 | 44 | 10 |
| 4 Buccal mucosa | 59 | 7 | 54 | 5 |
| 5 Floor of mouth and inferior surface of tongue | 136 | 19 | 130 | 6 |
| 6 Dorsum and borders of anterior two-thirds tongue | 150 | 32 | 115 | 35 |
| 7 Posterior third tongue (and whole tongue) | 75 | 15 | 70 | 5 |
| 8 Anterior faucial pillar and soft palate | 85 | 15 | 83 | 2 |
| 9 Tonsil proper | 41 | 8 | 34 | 7 |

*The age and duration of symptoms.*

It is well recognized that buccal cancer affects a relatively old group of patients and this series is no exception. This is the first fact that militates against long survival periods. It has not been so generally emphasized, however, that the duration of symptoms before treatment is often disconcertingly short. In spite of this many cases were of an advanced character, i.e. stages III or IV, when first seen. This is the second difficulty which makes the prognosis so comparatively bad.

Table II illustrates the age groups into which the patients fitted. None was under 35, and the oldest (a man with carcinoma of the lip) was 104.

TABLE II.—*Age Groups.*

| Age. | Number of cases (total 800). |
|---|---|
| Under 40 | 14 |
| 40–50 | 60 |
| 51–60 | 179 |
| 61–70 | 320 |
| 71 and over | 225 |
| Unrecorded | 2 |

Table III shows the duration of symptoms before the commencement of treatment. This did not vary much according to the site of the primary lesion. Patients with visible growths on the lip or front of the tongue did not attend for advice any sooner than those whose growths were situated further back, and in whom it was often the cervical metastases which were first noticed. It will be seen that two-thirds of the patients had a history of less than 6 months. This is in agreement with the figures of other investigators.

TABLE III.—*Duration of Symptoms before Treatment.*

| Duration of symptoms. | Number of cases (total 800). |
|---|---|
| Less than 3 months | 224 |
| 3–6 months | 307 |
| 7–12 months | 150 |
| 1–3 years | 68 |
| More than 3 years | 30 |
| Uncertain | 21 |

*The staging of the disease.*

Not all authorities agree on the staging of cancer of the mouth, although the presence or absence or regional lymph nodes is of course recognized as being of signal importance with regard to prognosis and treatment. We have adopted the following staging, which is similar to that employed at the Christie Hospital and Holt Radium Institute, Manchester :

*Stage I.*—Primary growth limited in extent to site of origin. No loss of mobility or function. No palpable nodes.

*Stage II.*—Primary growth spreading to adjacent structures. No palpable nodes.

*Stage III.*—Primary growth as in Stage I or II. Regional lymph nodes invaded, but mobile and confined to area of immediate lymphatic drainage (i.e. operable).

*Stage IV.*—Primary growth too advanced for any treatment. Lymph nodes fixed, matted or bilateral, or distant metastases present.

Table IV shows, as already mentioned, how many cases had already reached Stages III or IV when first seeking treatment. The lip, which is a notable exception, is charted separately. The fact that patients with cancer of the lip did not seek advice any sooner than those with growths at other sites confirms that cancer of the lip metastasizes more slowly than cancer inside the mouth.

TABLE IV.—*The Cases by Stages.*

| Stage. | Number of cases. | |
|---|---|---|
| | All sites except lip (total 640). | Lip (total 160). |
| I | 129 | 114 |
| II | 134 | 6 |
| III | 213 | 29 |
| IV | 162 | 11 |
| Unstaged | 2 | 0 |

Since the presence of involved lymph nodes so materially alters the prognosis it is convenient to summarize Table IV in the following manner :

| Stage. | All sites except lip. | Lip. |
|---|---|---|
| I and II | 263 (41%) | 120 (75%) |
| III and IV | 375 (59%) | 40 (25%) |

*Histology.*

Since the majority of the cases were treated by radiotherapy the whole neoplasm was very seldom available for histological study, but a biopsy was performed in about 4 out of every 5 cases, and the diagnosis of malignancy confirmed.

Broders' (1920) grading of neoplasms was given up several years ago at the Royal Cancer Hospital. It was recognized that wide variations in the type of cell could be encountered in different sections from the same growth, and that for this reason alone attempts at the estimation of the degree of differentiation from the biopsy of a small piece of tissue were likely to be not only of no value, but actually misleading.

Of the 800 cases under review a biopsy was performed in 639. The histology was as follows :

| | |
|---|---|
| Squamous carcinoma | 631 |
| Lymphoepithelioma | 4 |
| Basal-celled carcinoma | 1 |
| Malignant sialoma | 2 |
| Lymphosarcoma | 1 |
| | 639 |

*Wassermann reaction.*

This was performed on 500 out of the 800 cases. There were 79 positive and 421 negative results—a proportion of 16 per cent positive reactions. Growths of the tongue were most frequently associated with a positive Wassermann reaction, and in those patients upon whom the test was performed 28 per cent of positive reactions were recorded. Patients with cancer of the buccal mucosa had positive Wassermann reactions in 20 per cent of cases, while those with cancer of the palate and alveoli were seldom found to be positive (3 per cent and 0·5 per cent respectively).

*Multiple primaries.*

In 1932 Lord Moynihan recorded a case where a second primary had appeared in the mouth some years after the first growth had been successfully treated, and he added that he had never seen this occur before (Moynihan, 1932). This stimulated the collection of such cases, and Fraser (1931) recorded 5 multiple primaries in 105 cases of buccal cancer. It is now well recognized that such double or even triple primary growths may occur in different positions in the buccal cavity and upper air passages. Warren and Gates (1932) investigated the problem in considerable detail, and concluded that a patient with one cancer was statistically more likely to develop another cancer than a normal individual. Amongst their own cases they found 3·7 per cent of multiple primaries in 1078 patients dying from cancer. Watson (1939) calls attention to the fact that when a patient with carcinoma of the oesophagus has a second growth this is nearly always situated in the mouth. The corollary is also true, that when the first growth is in the mouth the second is very frequently in the upper digestive tract.

Amongst this series of 800 cases there were 30 patients who developed a second primary (3·75 per cent). The following criteria have been adopted for calling a neoplasm a new primary. It is recognized that these are not so strict as those proposed by other authorities, e.g. originally by Billroth (1889), but they seem to stand the test of practical experience.

Each growth must be distinct, and if arising in the same anatomical site (e.g. the buccal cavity) there must be no possibility of the one being a local recurrence of the other. Each growth must be proved histologically, and if the histology is the same (e.g. squamous-celled) then the possibility of metastasis must be reasonably excluded.

Table V shows the distribution of the 30 cases. These are grouped according to the site of the first and the subsequent growths. It will be seen that the majority of the latter occurred in the digestive tract, and most often between the lip and the cardiac sphincter of the stomach.

TABLE V.—*Multiple Primaries.*

| First primary. | | Subsequent primary. | | |
|---|---|---|---|---|
| | | Lip—cardia. | Cardia—rectum. | Other sites. |
| Lip | 16 | 8 | 1 | 7—6 skin<br>1 bladder |
| Tongue | 4 | 1 | 2 | 1— bladder |
| Rest of mouth | 10 | 3 | 2 | 5—1 skin<br>1 bronchus<br>1 nasopharynx<br>1 pyriform fossa<br>1 larynx |
| Total | 30 | 12 | 5 | 13 |

### RESULTS OF TREATMENT.

As previously stated, the majority of the cases were treated by radiotherapy. Many had a combination of different types of therapy, but it has been possible to analysé two reasonably large groups in detail—those treated by teleradium, occasionally assisted by a radium applicator, needles or radon seeds, and those treated by X-ray therapy.

### *Teleradium.*

The 5-gramme unit was used for the most part. A few cases were treated, mainly palliatively, with the 1-gramme unit. It will be observed in the account which follows that interstitial radium has been comparatively neglected. This was a deliberate decision made in order to determine the value of teleradium therapy.

### *X-ray therapy.*

This has included high voltage, medium voltage and intra-oral contact therapy. 400 kV. and 200 kV. tubes have been used to give external irradiation to the primary tumour and nodes. Usually the dose to the primary tumour has been increased by intra-oral contact therapy with a low voltage tube (140 kV., 60 kV., or 45 kV.). For Stage I and II cases, particularly cancer of the lip, low voltage contact therapy has sometimes been the only method of X-ray therapy employed.

In order to simplify the detailed tables of results, the primary sites have been condensed as follows :

| | | |
|---|---|---|
| Lip . . . . . . . . | | 160 cases (Table VI) |
| Anterior two-thirds of tongue . . . | | 150 cases (Table VII) |
| Other buccal sites : | | |
| Upper alveolus and hard palate . | 40 | 289 cases (Table VIII) |
| Lower alveolus . . . . . | 54 | |
| Buccal mucosa . . . . . | 59 | |
| Inferior surface of tongue and floor of mouth . . . . . . | 136 | |
| Oro-pharyngeal sites : | | |
| Posterior one-third of tongue . . | 75 | 201 cases (Table IX) |
| Anterior faucial pillar and soft palate | 85 | |
| Tonsil . . . . . . . | 41 | |
| | | 800 cases |

A simplified table indicating the survival rates for each individual site is given later (Table X).

*Definitions of terms used.*

*D.I.D.*—Dead of intercurrent disease without cancer present.

*Net number.*—The number of patients treated, less those untraced and those dead of intercurrent disease.

*Dead of cancer.*—Includes also those dead from unknown cause, intercurrent disease with cancer present, or following treatment, in most cases within three months of the completion of treatment.

*Net survival rate.*—The percentage of the net number of patients alive with or without cancer.

*Absolute survival rate.*—The percentage of the total number of new patients, including those not treated, who are still alive. This gives a true statistical picture of what becomes of all the patients who attend.

TABLE VI.—*Lip.* 160 *cases*—4 *not treated.*

| | Cases seen from 1936–45 inclusive : state at the end of 3 years, 156 cases. | | | | | | 1936–43 inclusive : 5 years, 122 cases. | | 1936–38 inclusive : 10 years, 42 cases. |
|---|---|---|---|---|---|---|---|---|---|
| Method of treatment :— | Radium. | | X-rays. | | All methods.* | | All methods. | | All methods. |
| Stage :— | I and II | III and IV | I and II | III and IV | I and II | III and IV | I and II | I II and IV | All stages. |
| Number treated . . . . | 39 | 10 | 74 | 21 | 120 | 36 | 93 | 29 | 42 |
| Untraced . . . . . | 1 | 2 | 12 | 1 | 14 | 3 | 21 | 3 | 9 |
| D.I.D. . . . . . | 2 | — | 6 | 2 | 9 | 2 | 10 | 1 | 9 |
| Net number . . . . | 36 | 8 | 56 | 18 | 97 | 31 | 62 | 25 | 24 |
| Dead of cancer . . . . | 6 | 5 | 5 | 12 | 11 | 18 | 12 | 18 | 14 |
| Alive . . . . . | 30 | 3 | 51 | 6 | 86 | 13 | 50 | 7 | 10 |
| Net survival rate . . | 83% | — | 91% | — | 89% | 42% | 81% | 28% | 42% |
| Absolute survival rate . | — | | — | | 62% | | 46% | | 23% |

* Including 12 treated by combined methods.

*The lip*—160 *cases.*

Of the 156 treated cases, 26 were treated by a radium applicator, 22 by teleradium with or without an applicator, 1 by radium needles, 95 by X-rays, and 12 by combined methods (Table VI).

The early cases responded best to X-ray therapy, and with this treatment a 3-year net survival rate of 91 per cent was obtained for Stage I and II cases. The considerable difference between the 3-year net and absolute survival rates is due to the rather large number of untraced cases.

*Anterior two-thirds of tongue*—150 *cases.*

Only those neoplasms occurring on the dorsum and lateral border of the tongue anterior to the circumvallate papillae are included in this group.

In this instance it is possible to compare the results of three methods of treatment :

*Radium.*—83 cases. Teleradium only was used in 67 cases, teleradium and interstitial needles in 12, needles only in 4. Many Stage I and II cases received what amounted to prophylactic irradiation of the lymphatic areas of the neck. It has already been stated that interstitial radium therapy was seldom used in order to give teleradium an extensive trial.

*X-rays.*—33 cases.

*Surgery only.*—14 cases. The method involved was usually diathermy excision of the primary neoplasm or of the anterior portion of the tongue, together with block dissection of the neck, either initially or at a later date when enlarged lymph nodes appeared.

*Combined methods.*—In addition combined treatments were given to 17 patients. Only 16 block dissections were performed in the whole group of 150 cases.

Table VII shows that in early cases surgery gave the best results (6 out of 7 living three years). The overall 3-year net survival rate for all methods of treatment was 30 per cent, the 5-year rate 20 per cent, and the 10 year rate 14 per cent.

TABLE VII.—*Anterior Two-thirds of Tongue.* 150 *cases*—3 *not treated.*

| | Cases seen from 1936–45 inclusive :— state at the end of 3 years, 147 cases. | | | | | | | | 1936–43 inclusive : 5 years, 128 cases. | | 1936–38 inclusive : 10 years, 42 cases. |
|---|---|---|---|---|---|---|---|---|---|---|---|
| Method of treatment :— | Radium. | | X-rays. | | Surgery only. | | All methods.* | | All methods. | | All methods. |
| Stage :— | I and II | III and IV | I and II | III and IV | I and II | III and IV | I and II | III and IV | I and II | III and IV | All stages. |
| Number treated | 43 | 40 | 15 | 18 | 9 | 5 | 77 | 70 | 66 | 62 | 42 |
| Untraced | 2 | — | — | 1 | — | 1 | 2 | 2 | 4 | 2 | 1 |
| D.I.D. | 4 | 1 | — | — | 2 | — | 8 | 1 | 9 | 2 | 5 |
| Net number | 37 | 39 | 15 | 17 | 7 | 4 | 67 | 67 | 53 | 58 | 36 |
| Dead of cancer | 22 | 38 | 7 | 13 | 1 | 4 | 34 | 60 | 36 | 53 | 31 |
| Alive | 15 | 1 | 8 | 4 | 6 | — | 33 | 7 | 17 | 5 | 5 |
| Net survival rate | — | | — | | — | | 49% | 10·5% | 32% | 9% | 14% |
| Absolute survival rate | — | | — | | — | | 27% | | 17% | | 11% |

* Including 17 treated by combined methods.

*The remainder of the buccal cavity*—289 *cases.*

This group includes neoplasms arising from the inferior surface of the tongue, floor of mouth, the mucosa covering the alveoli and the hard palate and that of the inner aspect of the cheek.

In the 163 cases treated with radium, teleradium only was used in 140 cases, teleradium combined with needles or an applicator in 17, and needles or an applicator in 6.

The results of radium treatment for Stage I and II cases are seen to be statistically better than those of X-ray treatment (Table VIII).

TABLE VIII.—*Upper Alveolus and Hard Palate, Lower Alveolus, Buccal Mucosa, Floor of Mouth and Inferior Surface of Tongue.* 289 *cases*—9 *not treated.*

| | Cases seen from 1936–45 inclusive :— state at the end of 3 years, 280 cases. | | | | | | 1936–43 inclusive : 5 years, 220 cases. | | 1936–38 inclusive : 10 years, 83 cases. |
|---|---|---|---|---|---|---|---|---|---|
| Method of treatment :— | Radium. | | X-rays. | | All methods.* | | All methods. | | All methods. |
| Stage :— | I and II | III and IV | I and II | III and IV | I and II | III and IV | I and II | III and IV | All stages. |
| Number treated | 63 | 100 | 41 | 47 | 121 | 159 | 86 | 134 | 83 |
| Untraced | 4 | 7 | 2 | 2 | 8 | 9 | 6 | 7 | 5 |
| D.I.D. | 3 | 1 | 2 | 3 | 5 | 6 | 7 | 8 | 8 |
| Net number | 56 | 92 | 37 | 42 | 108 | 144 | 73 | 119 | 70 |
| Dead of cancer | 20 | 77 | 25 | 36 | 50 | 120 | 45 | 103 | 64 |
| Alive | 36 | 15 | 12 | 6 | 58 | 24 | 28 | 16 | 6 |
| Net survival rate | — | | — | | 54% | 16·5% | 38% | 13·5% | 8·5% |
| Absolute survival rate | — | | — | | 28% | | 19·5% | | 7% |

* Including 29 treated by combined methods.

*Oro-pharyngeal sites*—201 *cases.*

This group has already been defined. Cases in which the whole tongue was involved have been included here with the posterior third of the tongue.

TABLE IX.—*Posterior One-third of Tongue (including Whole Tongue), Anterior Faucial Pillar and Soft Palate, Tonsil.* 201 *cases*—12 *not treated.*

| | Cases seen from 1936–45 inclusive :— state at the end of 3 years, 189 cases. | | | | | | 1936–43 inclusive : 5 years, 147 cases. | | 1936–38 inclusive : 10 years, 55 cases. |
|---|---|---|---|---|---|---|---|---|---|
| Method of treatment :— | Radium. | | X-rays. | | All methods.* | | All methods. | | All methods. |
| Stage :— | I and II | III and IV | I and II | III and IV | I and II | III and IV | I and II | III and IV | All stages. |
| Number treated | 33 | 70 | 13 | 49 | 58 | 131 | 44 | 103 | 55 |
| Untraced | — | 2 | — | 3 | — | 5 | 1 | 5 | 2 |
| D.I.D. | 5 | 3 | 1 | 2 | 6 | 5 | 9 | 5 | 5 |
| Net number | 28 | 65 | 12 | 44 | 52 | 121 | 34 | 93 | 48 |
| Dead of cancer | 15 | 52 | 6 | 39 | 27 | 102 | 22 | 86 | 41 |
| Alive | 13 | 13 | 6 | 5 | 25 | 19 | 12 | 7 | 7 |
| Net survival rate | — | | — | | 48% | 15·5% | 35% | 7·5% | 14·5% |
| Absolute survival rate | — | | — | | 22% | | 12% | | 11·5 |

* Including 24 treated by combined methods.

*Radium.*—103 cases. In the 103 cases treated with radium, teleradium only was used in 94, needles gave additional irradiation in 8 others, and 1 was treated solely by needling.

*X-rays.*—62 cases.

Although the numbers are small, Table IX suggests that the early cases responded equally well to X-ray therapy as to radium.

*Results for individual sites.*

It is of some interest to compare the response to treatment of each of the eight individual sites in the buccal cavity and oro-pharynx (the lip having already been considered). In order to keep Table X within comprehensible limits only the net survival rates are given. The numbers of cases for each particular site can be found by reference to earlier tables. The average figures are surprisingly uniform, namely, about 30 per cent 3-year, 20 per cent 5-year, and 10 per cent 10-year net survivals.

TABLE X.—*All Sites : Net Survival Rates,* 3, 5 *and* 10 *years.*
640 *cases*—24 *not treated.*

| | 1936–45 : 3 years. | | | 1936–43 : 5 years. | 1936–38 : 10 years. |
|---|---|---|---|---|---|
| Method of treatment :— | Radium. | X-rays. | All methods. | All methods. | All methods. |
| | % | % | % | % | % |
| Upper alveolus and hard palate | 43 | 20 | 30·5 | 19·5 | 10 |
| Lower alveolus | 28 | 31 | 30 | 27 | 16·5 |
| Buccal mucosa | 36 | 6 | 29·5 | 16·5 | 10 |
| Floor of mouth and inferior surface tongue | 34 | 30 | 36·5 | 25 | 3 |
| Anterior two-thirds tongue | 21 | 37 | 30 | 20 | 14 |
| Posterior one-third and whole tongue | 30 | 17 | 21·5 | 15 | 12 |
| Anterior pillar and soft palate | 28 | 20 | 27 | 13 | 11 |
| Tonsil | 24 | 28 | 29·5 | 19 | 23 |
| All sites | 29 | 24·5 | 30 | 20 | 11·5 |

*Block dissection.*

The total number of block dissections performed was very small. During the period under review the idea of prophylactic block dissection was being abandoned, and operation performed only when operable lymph nodes were

TABLE XI.—*Block Dissections.*

| Site :— | Lip. | Anterior two-thirds of tongue. | Other sites. | Total. |
|---|---|---|---|---|
| Number | 7 | 16 | 18 | 41 |
| Operation part of first planned treatment | 2 | 10 | 10 | 22 |
| Operation part of subsequent treatment | 5 | 6 | 8 | 19 |
| Section positive | 4 | 9 | 12 | 25 |
| Section negative | 3 | 7 | 6 | 16 |
| Operation deaths | 0 | 2 | 3 | 5=12% |

palpable. Since only a few of these operations were on patients who had had no previous irradiation to the neck, it is not possible to contrast the relative efficiency of surgery and radiation as a means of treating secondary lymph nodes.

*Evaluation of First Planned Treatment.*

We have already stated that a scheme of treatment is decided upon when the patient is first seen. It is therefore of interest to know how often this treatment succeeds or fails in its objective, and in the latter case, when there is a recurrence, what are the chances of further arresting the disease.

These facts cannot be determined with any real accuracy, since each case clearly has to be assessed on its clinical development and course. Some cases can be excluded when first seen as being obviously incurable, and we are not concerned here with the palliative effects of treatment, however beneficial to the patient these may be.

We have selected only those cases in whom the initial treatment has been successful to the point of causing regression of the primary (in Stages I and II cases), or of the primary and secondary (in Stages III and IV cases).

Tables XII and XIII give some idea of the subsequent clinical history of those patients whose disease is apparently controlled by the first treatment instituted. Three years have been arbitrarily chosen as the period for which success or failure of the first treatment has been calculated, because the great majority of recurrences from cancer of the mouth occur during this period.

TABLE XII.—*Results of First Planned Treatments.*

| Site :— | Lip. | | Mouth. | |
|---|---|---|---|---|
| Stage :— | I II | III IV | I II | III IV |
| Complete regression of disease following first treatment | 90 | 29 | 256 | 248 |
| No recurrences within the first 3 years | 70 | 10 | 108 | 45 |
| Recurrences within the first 3 years | 20 (22%) | 19 (65%) | 148 (58%) | 203 (82%) |

Many of those who recurred within the first three years were given subsequent treatment to the recurrences. The number of those cases thus re-treated who survived another 3 years is given in Table XIII. It is clearly shown that except

TABLE XIII.—*Results of Treatment to Recurrences.*

| Site :— | Lip. | | Mouth. | |
|---|---|---|---|---|
| Stage :— | I II | III IV | I II | III IV |
| Number of recurrences within the first 3 years | 20 | 19 | 148 | 203 |
| Number re-treated | 20 | 19 | 71 | 86 |
| Number successfully re-treated 3 years no recurrence | 10 (50%) | 0 | 19 (13%) | 14 (7%) |

in Stage I and II cancers of the lip, re-treatment is rarely successful. If the first planned treatment fails, the patient does not often have a second chance.

*Response of Primary and Secondary to Irradiation.*

Glücksmann (1948) has stated that the response to irradiation of the primary and secondary manifestations of cancer of the mouth is about the same, and that those growths "with a tendency to lymph-node involvement differ in their biology and radio-curability from those without such a tendency." The more generally held view is that while the primary may be "cured," the secondary is often more resistant to treatment.

Only Stage III and IV cases can be analysed if the theory that an irradiated primary will recur as frequently as an irradiated secondary is to be examined.

There were 248 patients with Stage III and IV growths arising from buccal sites in whom radiotherapy produced regression of both primary and secondary growths. Forty-five remained well, and 203 recurred : 61 in the primary only, 69 in the secondary only, 73 in both. We can say then that recurrence took place in the primary 134 times and in the secondary 142 times— a difference which is only very slight. In considering the 73 cases where recurrence took place in primary and secondary, it is possible to argue that the recurrence may have occurred first in the primary, which then metastasized for the second time to lymph nodes, thus producing apparent recurrence in the secondary. If this were so there would have been 134 recurrences in the primary against only 69 in the secondary. Glücksmann's observations therefore appear to be confirmed, and it may be that recurrence in the primary is actually more common than recurrence in the secondary.

COMPARISON OF RESULTS OF TREATMENT.

It is difficult to draw conclusions from comparison of the statistics of different centres and authorities unless the material as well as the results can be compared. Owing to the lack of agreed criteria as regards the staging and position of the primary neoplasm accurate statistical comparison is impossible.

TABLE XIV.—*5-year Net Survival Rates.*

| | Holt Radium Institute. 1934–38 : 1250 cases.* | | | | Royal Cancer Hospital. 1936–43 : 617 cases.† | | | | Royal Cancer Hospital 5-year survival figures adjusted to compare with Holt Radium Institute.‡ |
|---|---|---|---|---|---|---|---|---|---|
| | Numbe r of cases. | Stages I and II. | Stages III and IV. | 5-year survival all stages. | Number of cases. | Stages I and II. | Stages III and IV. | 5-year survival all stages. | |
| | | % | % | % | | % | % | % | % |
| Lip | 175 | 84 | 16 | **65** | 122 | 76 | 24 | **67** | **73** |
| Whole tongue | 359 | 62 | 38 | **25** | 180 | 43 | 57 | **18** | **25** |
| Buccal sites | 407 | 57 | 43 | **33** | 220 | 39 | 61 | **23** | **27** |
| Oro-phar' sites | 309 | 42 | 58 | **17** | 95 | 35 | 65 | **15** | **17** |

* Tables XXXIV and XLV, 1946 Report. † Tables VI, VII, VIII and IX.

‡ Dr. H. O. Hartley has kindly done a significance test on these figures. The number of cases has been adjusted so that the proportion of Stage III and IV Royal Cancer Hospital patients is the same as that of the Holt Radium Institute series. He states that all differences are statistically insignificant.

Certainly there is no reason for complacency. Butlin's (1908) figure of 28 per cent 3-year survivals for the surgical treatment of cancer of the tongue between 1880 and 1908 (197 cases) still bears comparison with the figures of 40 years later.

The method of recording results at the Holt Radium Institute at Manchester (1946) approximates most closely to that of the Royal Cancer Hospital, and Table XIV gives an accurate comparison of results between these two centres. It will be seen at once that the Holt Radium Institute in 5 years had twice as many cases as the Royal Cancer Hospital had in 8 years. It should also be noted that they were fortunate in getting their patients at a considerably earlier clinical stage than other centres. In the case of the anterior two-thirds of the tongue, Stage III and IV cases numbered 48 per cent at the Royal Cancer Hospital, as against 38 per cent at the Holt Radium Institute. At the Westminster Hospital Cade (1949) reported almost 60 per cent with palpable nodes when first seen, and at the Radiumhemmet Jacobsson (1948) reported 48 per cent.

Table XV is an attempt to compare the published figures of five different authorities. They have been selected as offering the widest difference in methods of treatment, and the following brief summary explains how this is so:

*Royal Cancer Hospital.*—Roughly two-thirds of the cases of buccal and oro-pharyngeal cancer have been treated by teleradium and one-third by high voltage X-ray therapy. Twice as many patients with lip cancer have been treated by low voltage X-ray therapy as by radium. Surgery has seldom been used even in the treatment of cervical lymph node metastases.

*Holt Radium Institute* (Manchester, 1946).—Radium implants (planar and volume) were used for the treatment of the primary growth in 80 per cent of

TABLE XV.—*5-year Survivals : All Stages.*

* The figures from the Royal Cancer Hospital and from the Holt Radium Institute are net survival rates. Owing to differences in methods of recording, the figures from the other centres are not strictly comparable.

buccal and faucial cases, and high voltage X-ray therapy in the other 20 per cent. Teleradium was not employed. Almost all lip cancers were treated by a radium mould or needles. Block dissection was done on mobile cervical metastases, X-rays or radium treatment given to the inoperable cases.

*Westminster Hospital* (Cade, 1949).—The figures in the table are taken from Sir Stanford Cade's book. All forms of radium therapy have been employed, but with the emphasis on interstitial needling for the accessible growths of the mouth and lip, and teleradium for the oro-pharyngeal primaries. For cervical metastases block dissection has been the method of choice, or radium therapy for the inoperable cases. X-rays have been used comparatively rarely.

*Memorial Hospital, New York* (Martin, 1948).—Dr. Hayes Martin states that surgical excision is the method of choice for cancer of the lip, gum, anterior portion of the tongue, cheek, floor of mouth or palate. For oro-pharyngeal growths a combination of roentgen therapy and interstitial radiation is indicated. Cervical metastases are treated by dissection when operable and by X-rays when not.

*Radiumhemmet, Stockholm* (Berven, 1937).—For more than 20 years the usual treatment for growths of the tongue and most other buccal sites has been teleradium, with diathermy coagulation of any residual mass. The neck has been treated by teleradium, followed, if necessary, by block dissection.

### SUMMARY.

Eight hundred cases of cancer of the buccal cavity treated at the Royal Cancer Hospital between 1936 and 1945 are presented and analysed. These are grouped in a manner which seems to satisfy the anatomical, clinical and therapeutic aspects of the disease most conveniently.

Seventy per cent of patients were over 60, and ten times as many men were affected as women. Sixty-five per cent had symptoms for less than 6 months before treatment commenced, yet (with the exception of carcinoma of the lip) in 60 per cent of cases the disease had already metastasized to the cervical lymph nodes when first seen. The treatment of these 800 cases has been largely by radiotherapy.

Comparison of the results of treatment published by different authorities is difficult because of lack of uniformity in criteria adopted. Table XV shows wide divergencies for instance in the survivals of patients with growths of the buccal mucosa, and to a lesser extent of the alveoli and palate. This is largely due to the small numbers of the cases.

The last section of this paper shows that although very different methods of treatment may be employed, the results on the whole are much the same. It is therefore inadvisable to attempt definite conclusions from the analysis of the cases presented. The following views are therefore put forward only tentatively :

Carcinoma of the lip is best treated by superficial X-ray therapy.

Small Stage I primary growths in accessible situations within the mouth may be widely excised, but can be treated with equal success by radiotherapy.

The patient should be seen in the follow-up department every month in order that any recurrence in the primary or metastasis in the cervical lymph nodes may be speedily attacked.

Recurrence in the primary is more common than is usually appreciated, and may be as frequent as recurrences in the nodes after both have been apparently successfully treated.

In most buccal and oro-pharyngeal sites the results of teleradium therapy are superior to those of X-ray therapy.

The teleradium treatment of growths of the anterior two-thirds of the tongue has not been so successful as the interstitial methods employed elsewhere.

This paper is based on a report prepared at the direction of the Clinical Research Sub-Committee of the Royal Cancer Hospital. The cases have been under the care of all members of the staff of the hospital, past and present, to whom grateful acknowledgment is made for permission to publish them. In the preparation of the paper much help was derived from Copeland-Chatterton punch cards, many of which had been completed by Dr. J. C. Dore, and from Hollerith cards. The latter system of record keeping was adopted by the Royal Cancer Hospital in 1945.

### REFERENCES.

BERVEN, E. G. E.—(1937) *Acta Radiol.*, **18**, 16.
BILLROTH, T.—(1889) 'General Surgery, Pathology and Therapeutics.' Trans. by C. E. Hackley. New York (Appleton).
BRODERS, A. C.—(1920) *J. Amer. med. Ass.*, **74**, 656.
BUTLIN, H. T.—(1908) 'On the Results of Operations for Carcinoma of the Tongue.' London (Adlard & Son).
CADE, S.—(1949) 'Malignant Disease and its Treatment by Radium,' 2nd ed., Vol. 2. Bristol (John Wright & Sons).
FRASER, J.—(1931) *Newcastle med. J.*, ii, 53.
GLÜCKSMANN, A.—(1948) *Brit. J. Radiol.*, **21**, 559.
HOLT RADIUM INSTITUTE.—(1946) 2nd Statistical Report.
JACOBSSON, F.—(1948) *Acta Radiol.*, Suppl. 68.
MARTIN, H. E.—(1948) 'Cancer of the Head and Neck.' New York (American Cancer Society).
MOYNIHAN, B. G. A.—(1932) *Lancet*, i, 19.
STOCKHOLM CANCER SOCIETY.—(1949) Report on Cases Treated at the Radiumhemmet, 1921–1947.
WARREN, S., AND GATES, O.—(1932) *Amer. J. Cancer*, **16**, 1358.
WATSON, W. L.—(1939) *Amer. J. Roentgenol.*, **41**, 420.